# Ethyl 8,13-dioxa-21-aza­penta­cyclo­[18.5.1.0^2,7^.0^14,19^.0^21,25^]hexa­cosa-2(7),3,5,14,16,18-hexa­ene-26-carboxyl­ate

**DOI:** 10.1107/S1600536812049094

**Published:** 2012-12-05

**Authors:** Sibi Narayanan, Thothadri Srinivasan, Santhanagopalan Purushothaman, Raghavachary Raghunathan, Devadasan Velmurugan

**Affiliations:** a Centre of Advanced Study in Crystallography and Biophysics, University of Madras, Guindy Campus, Chennai 600 025, India; b Department of Organic Chemistry, University of Madras, Guindy Campus, Chennai 600 025, India

## Abstract

In the title compound, C_26_H_31_NO_4_, the five-membered rings of the central pyrrolizine system adopt N-envelope conformations. The ethyl acetate group adopts an extended conformation. The dihedral angle between the benzene rings is 36.6 (1)°. In the crystal, C—H⋯O hydrogen bonds form a zigzag chain running along the *b-*axis directions. The crystal structure is futher consolidated by C—H⋯π inter­actions.

## Related literature

For the biological activity of pyrrolidine derivatives, see: Pinna *et al.* (2002 ▶); Araki *et al.* (2002 ▶). For a related structure, see: Nirmala *et al.* (2008 ▶).
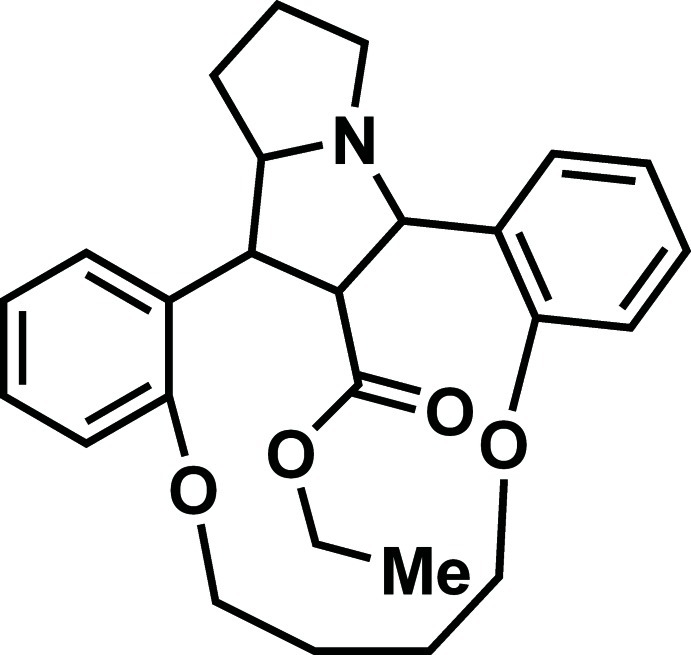


## Experimental

### 

#### Crystal data

C_26_H_31_NO_4_
*M* *_r_* = 421.52
Monoclinic, 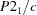

*a* = 10.4784 (5) Å
*b* = 10.2624 (4) Å
*c* = 21.0937 (10) Å
β = 95.350 (3)°
*V* = 2258.40 (18) Å^3^
*Z* = 4
Mo *K*α radiation
μ = 0.08 mm^−1^
*T* = 293 K
0.25 × 0.22 × 0.19 mm

#### Data collection

Bruker APEXII CCD area-detector diffractometer
Absorption correction: multi-scan (*SADABS*; Bruker, 2008 ▶) *T*_min_ = 0.979, *T*_max_ = 0.984
20978 measured reflections
5598 independent reflections
3092 reflections with *I* > 2σ(*I*)
*R*_int_ = 0.031

#### Refinement

*R*[*F* ^2^ > 2σ(*F* ^2^)] = 0.057
*wR*(*F* ^2^) = 0.180
*S* = 1.02
5598 reflections
281 parameters
H-atom parameters constrained
Δρ_max_ = 0.35 e Å^−3^
Δρ_min_ = −0.23 e Å^−3^

### 

Data collection: *APEX2* (Bruker, 2008 ▶); cell refinement: *SAINT* (Bruker, 2008 ▶); data reduction: *SAINT*; program(s) used to solve structure: *SHELXS97* (Sheldrick, 2008 ▶); program(s) used to refine structure: *SHELXL97* (Sheldrick, 2008 ▶); molecular graphics: *ORTEP-3* (Farrugia, 2012 ▶); software used to prepare material for publication: *SHELXL97* and *PLATON* (Spek, 2009 ▶).

## Supplementary Material

Crystal structure: contains datablock(s) global, I. DOI: 10.1107/S1600536812049094/pv2608sup1.cif

Structure factors: contains datablock(s) I. DOI: 10.1107/S1600536812049094/pv2608Isup2.hkl

Supplementary material file. DOI: 10.1107/S1600536812049094/pv2608Isup3.cml

Additional supplementary materials:  crystallographic information; 3D view; checkCIF report

## Figures and Tables

**Table 1:** Hydrogen-bond geometry (Å, °) *Cg*3 and *Cg*4 are the centroids of the C8–C13 and C18–C23 rings, respectively.

*D*—H⋯*A*	*D*—H	H⋯*A*	*D*⋯*A*	*D*—H⋯*A*
C11—H11⋯O4^i^	0.93	2.60	3.419 (3)	148
C17—H17*B*⋯*Cg*4^ii^	0.97	2.97	3.817 (3)	146
C22—H22⋯*Cg*3^iii^	0.93	2.94	3.770 (3)	150

Symmetry codes: (i) 
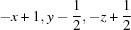
; (ii) 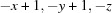; (iii) 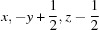.

**Fig. 1.**
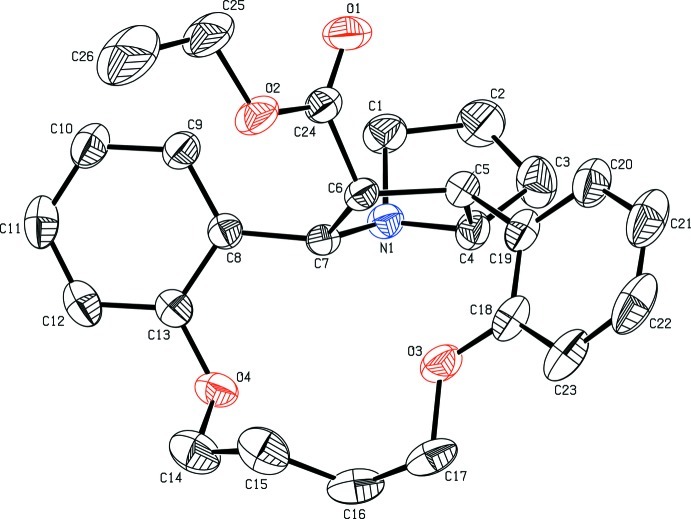
The molecular structure of the title compound, showing displacement ellipsoids drawn at the 30% probability level. H atoms have been omitted for clarity.

**Fig. 2.**
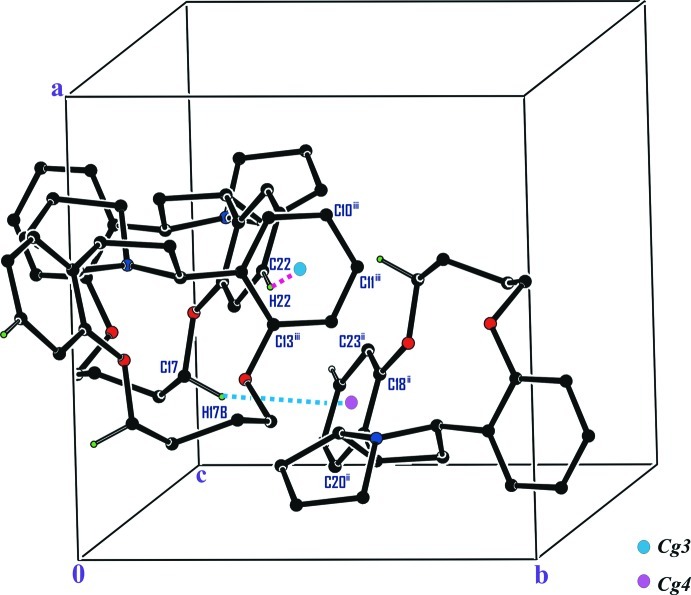
The crystal structure showing the C—H···π interactions. The ethyl acetate group is omitted for clarity.

**Fig. 3.**
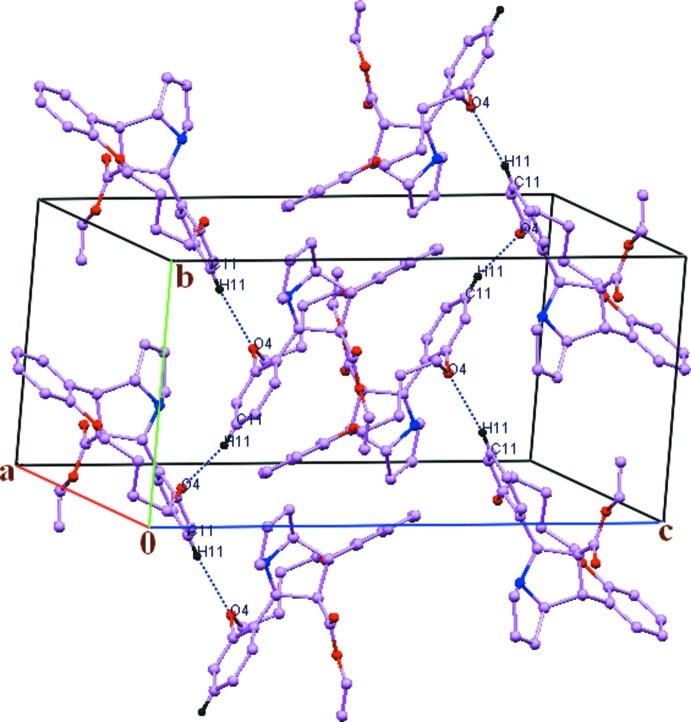
Molecular packing of the title compound, in which hydrogen bonds forms a zigzag chain running along b axis. For the sake of clarity, the H atoms not involved in hydrogen bonds have been omitted.

## supplementary crystallographic information

### Comment

Pyrrolidine derivatives are widely used as organic catalysts and serve as important structural units in biologically active molecules (Pinna *et al.*, 2002). The spiro-pyrrolidine ring system is also associated with antitumour activity (Araki *et al.*, 2002). In continuation of our work on the crystal structure analysis of spiro-pyrrolidine derivatives, the crystal structure of the title compound has been carried out and the results are presented here.

In the title compound (Fig. 1) the bond lengths and angles are comparable to the corresponding values as observed in a related structure (Nirmala *et al.*, 2008). The pyrrolizine ring system is folded about the bridging N1—C4 bond, as observed in related structure (Nirmala *et al.*, 2008). The two benzene rings (C8—C13 and C18—C23) make dihedral angles of 71.7 (1)° and 55.3 (1)°, respectively, with respect to the pyrrolizine ring system; the dihedral angle between the two benzene rings is 36.6 (1)°. The dimethoxybutane group connects these two benzene rings at *meta* positions (at atom C13 and C18). The ethyl acetate group adopts an extended conformation as can be seen from the torsion angle [C6—C24—O2—C25 = -175.1 (2)°]. The atom O3 lies in the plane of the benzene ring (C18–C23) (deviation -0.008 (1) Å) while O4 deviates by 0.130 (1) Å from the least squares plane of the benzene ring (C8–C13). In the pyrrolizine ring system, both of the rings (N1/C1—C4) and (N1/C4—C7) adopt N1-envelope conformations, with N1 0.505 (3) and 0.566 (3) Å from the least-squres planes of the remaining rings atoms, respevtively. The crystal structure is stabilized by C—H···π interactions (Fig. 2). In the crystal structure, the intermolecular C11—H11···O4 hydrogen bonds form a zigzag chain running along the *b*-axis (Fig. 3).

### Experimental

A solution of (*E*)-ethyl 3-(2-(4-(2-formylphenoxy)butoxy)phenyl)acrylate (200 mg, 0.54 mmol) and *L*-proline (75 mg, 0.65 mmol) was refluxed in dry toluene under N_2_ atmosphere for 12 h under Dean-Stark apparatus. After the completion of reaction as indicated by TLC, toluene was evaporated under reduced pressure. The crude product was washed with water and extracted with dichloromethane (4x20 mL). The combined organic layers were dried (MgSO_4_) and filtered, concentrated in vacuum. The crude product was purified by column chromatography using hexane: EtOAc (8:2) as an eluent. The product was dissolved in ethylacetate and heated for two minutes. The resulting solution was subjected to crystallization by slow evaporation of the solvent resulting in single crystals suitable for XRD studies.

### Refinement

All H atoms were fixed geometrically and allowed to ride on their parent C atoms, with C—H distances fixed in the range 0.93–0.97 Å with *U*_iso_(H) = 1.5*U*_eq_(methyl C) or 1.2*U*_eq_(non-methyl C) atoms.

### Crystal data

**Table:**

C_26_H_31_NO_4_	*F*(000) = 904
*M**_r_* = 421.52	*D*_x_ = 1.240 Mg m^−^^3^
Monoclinic, *P*2_1_/*c*	Mo *K*α radiation, λ = 0.71073 Å
Hall symbol: -P 2ybc	Cell parameters from 5598 reflections
*a* = 10.4784 (5) Å	θ = 1.9–28.3°
*b* = 10.2624 (4) Å	µ = 0.08 mm^−^^1^
*c* = 21.0937 (10) Å	*T* = 293 K
β = 95.350 (3)°	Block, colorless
*V* = 2258.40 (18) Å^3^	0.25 × 0.22 × 0.19 mm
*Z* = 4	

### Data collection

**Table:**

Bruker APEXII CCD area-detector diffractometer	5598 independent reflections
Radiation source: fine-focus sealed tube	3092 reflections with *I* > 2σ(*I*)
Graphite monochromator	*R*_int_ = 0.031
ω and φ scans	θ_max_ = 28.3°, θ_min_ = 1.9°
Absorption correction: multi-scan (*SADABS*; Bruker, 2008)	*h* = −13→11
*T*_min_ = 0.979, *T*_max_ = 0.984	*k* = −13→13
20978 measured reflections	*l* = −25→28

### Refinement

**Table:**

Refinement on *F*^2^	Primary atom site location: structure-invariant direct methods
Least-squares matrix: full	Secondary atom site location: difference Fourier map
*R*[*F*^2^ > 2σ(*F*^2^)] = 0.057	Hydrogen site location: inferred from neighbouring sites
*wR*(*F*^2^) = 0.180	H-atom parameters constrained
*S* = 1.02	*w* = 1/[σ^2^(*F*_o_^2^) + (0.0815*P*)^2^ + 0.4765*P*] where *P* = (*F*_o_^2^ + 2*F*_c_^2^)/3
5598 reflections	(Δ/σ)_max_ < 0.001
281 parameters	Δρ_max_ = 0.35 e Å^−^^3^
0 restraints	Δρ_min_ = −0.23 e Å^−^^3^

### Special details

**Table:**

Geometry. All e.s.d.'s (except the e.s.d. in the dihedral angle between two l.s. planes) are estimated using the full covariance matrix. The cell e.s.d.'s are taken into account individually in the estimation of e.s.d.'s in distances, angles and torsion angles; correlations between e.s.d.'s in cell parameters are only used when they are defined by crystal symmetry. An approximate (isotropic) treatment of cell e.s.d.'s is used for estimating e.s.d.'s involving l.s. planes.
Refinement. Refinement of *F*^2^ against ALL reflections. The weighted *R*-factor *wR* and goodness of fit *S* are based on *F*^2^, conventional *R*-factors *R* are based on *F*, with *F* set to zero for negative *F*^2^. The threshold expression of *F*^2^ > σ(*F*^2^) is used only for calculating *R*-factors(gt) *etc*. and is not relevant to the choice of reflections for refinement. *R*-factors based on *F*^2^ are statistically about twice as large as those based on *F*, and *R*- factors based on ALL data will be even larger.

### Fractional atomic coordinates and isotropic or equivalent isotropic displacement parameters (Å^2^)

**Table:**

	*x*	*y*	*z*	*U*_iso_*/*U*_eq_	
C1	0.8192 (2)	0.3134 (2)	0.22908 (12)	0.0675 (6)	
H1A	0.8905	0.2679	0.2129	0.081*	
H1B	0.8112	0.2856	0.2725	0.081*	
C2	0.8361 (3)	0.4599 (3)	0.22534 (16)	0.0992 (10)	
H2A	0.8050	0.5013	0.2623	0.119*	
H2B	0.9261	0.4812	0.2245	0.119*	
C3	0.7632 (3)	0.5066 (2)	0.16699 (14)	0.0835 (8)	
H3A	0.8199	0.5460	0.1386	0.100*	
H3B	0.6999	0.5705	0.1769	0.100*	
C4	0.6989 (2)	0.38673 (18)	0.13682 (10)	0.0549 (5)	
H4	0.6104	0.4070	0.1207	0.066*	
C5	0.7702 (2)	0.32113 (19)	0.08370 (10)	0.0530 (5)	
H5	0.8609	0.3446	0.0914	0.064*	
C6	0.75954 (17)	0.17292 (18)	0.09547 (9)	0.0456 (4)	
H6	0.7077	0.1345	0.0591	0.055*	
C7	0.67976 (17)	0.16759 (17)	0.15509 (9)	0.0448 (4)	
H7	0.5906	0.1727	0.1366	0.054*	
C8	0.68313 (18)	0.04520 (18)	0.19466 (9)	0.0485 (5)	
C9	0.7948 (2)	−0.0100 (2)	0.22369 (11)	0.0618 (6)	
H9	0.8724	0.0329	0.2213	0.074*	
C10	0.7937 (2)	−0.1271 (2)	0.25608 (11)	0.0709 (6)	
H10	0.8698	−0.1622	0.2749	0.085*	
C11	0.6808 (3)	−0.1907 (2)	0.26029 (11)	0.0721 (7)	
H11	0.6803	−0.2705	0.2811	0.087*	
C12	0.5678 (2)	−0.1378 (2)	0.23405 (11)	0.0669 (6)	
H12	0.4908	−0.1808	0.2380	0.080*	
C13	0.56846 (19)	−0.02010 (19)	0.20165 (10)	0.0520 (5)	
C14	0.3735 (3)	−0.0270 (3)	0.13226 (14)	0.0883 (8)	
H14A	0.2849	−0.0179	0.1414	0.106*	
H14B	0.3944	−0.1191	0.1327	0.106*	
C15	0.3897 (3)	0.0281 (3)	0.06888 (13)	0.0850 (8)	
H15A	0.3445	−0.0267	0.0367	0.102*	
H15B	0.4800	0.0259	0.0621	0.102*	
C16	0.3404 (2)	0.1700 (3)	0.05960 (13)	0.0819 (8)	
H16A	0.2478	0.1684	0.0516	0.098*	
H16B	0.3618	0.2181	0.0987	0.098*	
C17	0.3953 (2)	0.2410 (3)	0.00565 (12)	0.0754 (7)	
H17A	0.3845	0.1895	−0.0330	0.090*	
H17B	0.3524	0.3240	−0.0021	0.090*	
C18	0.6024 (2)	0.3342 (2)	−0.01145 (10)	0.0632 (6)	
C19	0.7248 (2)	0.3648 (2)	0.01703 (10)	0.0590 (5)	
C20	0.8025 (3)	0.4390 (2)	−0.01830 (13)	0.0795 (7)	
H20	0.8837	0.4618	−0.0002	0.095*	
C21	0.7642 (4)	0.4804 (3)	−0.07941 (16)	0.1016 (11)	
H21	0.8190	0.5300	−0.1020	0.122*	
C22	0.6452 (4)	0.4479 (3)	−0.10624 (14)	0.1009 (11)	
H22	0.6191	0.4750	−0.1475	0.121*	
C23	0.5625 (3)	0.3748 (3)	−0.07280 (12)	0.0836 (8)	
H23	0.4813	0.3533	−0.0913	0.100*	
C24	0.8885 (2)	0.1065 (2)	0.10175 (10)	0.0535 (5)	
C25	0.9933 (3)	−0.0951 (3)	0.08828 (19)	0.1080 (11)	
H25A	1.0491	−0.0726	0.0558	0.130*	
H25B	1.0390	−0.0777	0.1295	0.130*	
C26	0.9634 (5)	−0.2220 (4)	0.0838 (3)	0.211 (3)	
H26A	0.9064	−0.2437	0.1153	0.316*	
H26B	1.0401	−0.2731	0.0908	0.316*	
H26C	0.9222	−0.2401	0.0422	0.316*	
N1	0.70022 (15)	0.29156 (15)	0.18853 (8)	0.0499 (4)	
O1	0.98902 (15)	0.15226 (17)	0.12248 (9)	0.0811 (5)	
O2	0.87743 (14)	−0.01498 (15)	0.08036 (8)	0.0720 (5)	
O3	0.52806 (14)	0.26037 (15)	0.02487 (7)	0.0661 (4)	
O4	0.45450 (13)	0.03858 (14)	0.17936 (7)	0.0621 (4)	

### Atomic displacement parameters (Å^2^)

**Table:**

	*U*^11^	*U*^22^	*U*^33^	*U*^12^	*U*^13^	*U*^23^
C1	0.0656 (14)	0.0668 (14)	0.0663 (14)	0.0016 (11)	−0.0136 (11)	−0.0109 (12)
C2	0.097 (2)	0.0708 (17)	0.121 (2)	−0.0092 (15)	−0.0377 (18)	−0.0207 (17)
C3	0.109 (2)	0.0525 (13)	0.0868 (18)	−0.0092 (13)	−0.0011 (16)	−0.0116 (13)
C4	0.0623 (12)	0.0417 (10)	0.0593 (12)	0.0048 (9)	−0.0021 (10)	−0.0014 (9)
C5	0.0510 (11)	0.0489 (11)	0.0587 (12)	0.0030 (9)	0.0023 (9)	0.0060 (9)
C6	0.0431 (10)	0.0465 (10)	0.0460 (10)	0.0047 (8)	−0.0023 (8)	0.0008 (8)
C7	0.0373 (9)	0.0449 (10)	0.0514 (11)	0.0019 (7)	0.0004 (8)	−0.0001 (8)
C8	0.0492 (11)	0.0465 (10)	0.0503 (11)	0.0015 (8)	0.0078 (8)	−0.0012 (8)
C9	0.0551 (12)	0.0651 (13)	0.0656 (13)	0.0085 (10)	0.0073 (10)	0.0154 (11)
C10	0.0783 (16)	0.0695 (15)	0.0662 (14)	0.0199 (13)	0.0133 (12)	0.0177 (12)
C11	0.107 (2)	0.0504 (12)	0.0629 (14)	0.0079 (13)	0.0286 (14)	0.0091 (11)
C12	0.0791 (16)	0.0552 (13)	0.0706 (15)	−0.0138 (12)	0.0285 (12)	−0.0063 (11)
C13	0.0549 (12)	0.0489 (11)	0.0537 (12)	−0.0024 (9)	0.0131 (9)	−0.0100 (9)
C14	0.0753 (17)	0.0911 (19)	0.097 (2)	−0.0247 (14)	0.0025 (15)	−0.0211 (16)
C15	0.0890 (19)	0.0872 (19)	0.0787 (18)	−0.0212 (15)	0.0070 (14)	−0.0342 (15)
C16	0.0521 (13)	0.119 (2)	0.0720 (16)	0.0111 (14)	−0.0090 (11)	−0.0198 (16)
C17	0.0604 (14)	0.0939 (18)	0.0671 (15)	0.0243 (13)	−0.0197 (12)	−0.0130 (14)
C18	0.0819 (16)	0.0564 (13)	0.0509 (12)	0.0268 (11)	0.0040 (11)	0.0060 (10)
C19	0.0708 (14)	0.0510 (11)	0.0561 (12)	0.0179 (10)	0.0105 (10)	0.0085 (10)
C20	0.0900 (18)	0.0721 (16)	0.0801 (17)	0.0206 (13)	0.0275 (14)	0.0236 (13)
C21	0.127 (3)	0.098 (2)	0.087 (2)	0.045 (2)	0.045 (2)	0.0428 (17)
C22	0.145 (3)	0.099 (2)	0.0618 (17)	0.060 (2)	0.0245 (19)	0.0282 (16)
C23	0.109 (2)	0.0799 (17)	0.0597 (15)	0.0417 (16)	−0.0056 (14)	0.0068 (13)
C24	0.0504 (12)	0.0560 (12)	0.0544 (12)	0.0093 (10)	0.0058 (9)	0.0045 (10)
C25	0.0796 (19)	0.082 (2)	0.160 (3)	0.0404 (15)	0.0002 (19)	−0.009 (2)
C26	0.146 (4)	0.091 (3)	0.388 (10)	0.064 (3)	−0.015 (5)	−0.020 (4)
N1	0.0486 (9)	0.0483 (9)	0.0519 (9)	0.0028 (7)	−0.0004 (7)	−0.0038 (7)
O1	0.0442 (9)	0.0862 (12)	0.1114 (14)	0.0063 (8)	−0.0005 (9)	−0.0067 (10)
O2	0.0611 (10)	0.0598 (9)	0.0935 (12)	0.0227 (7)	−0.0021 (8)	−0.0092 (8)
O3	0.0603 (9)	0.0730 (10)	0.0612 (9)	0.0098 (7)	−0.0146 (7)	0.0074 (8)
O4	0.0460 (8)	0.0626 (9)	0.0775 (10)	−0.0076 (7)	0.0047 (7)	−0.0152 (7)

### Geometric parameters (Å, º)

**Table:**

C1—N1	1.462 (3)	C14—O4	1.415 (3)
C1—C2	1.516 (4)	C14—C15	1.476 (4)
C1—H1A	0.9700	C14—H14A	0.9700
C1—H1B	0.9700	C14—H14B	0.9700
C2—C3	1.467 (4)	C15—C16	1.552 (4)
C2—H2A	0.9700	C15—H15A	0.9700
C2—H2B	0.9700	C15—H15B	0.9700
C3—C4	1.514 (3)	C16—C17	1.509 (4)
C3—H3A	0.9700	C16—H16A	0.9700
C3—H3B	0.9700	C16—H16B	0.9700
C4—N1	1.463 (3)	C17—O3	1.427 (3)
C4—C5	1.556 (3)	C17—H17A	0.9700
C4—H4	0.9800	C17—H17B	0.9700
C5—C19	1.510 (3)	C18—O3	1.371 (3)
C5—C6	1.547 (3)	C18—C23	1.386 (3)
C5—H5	0.9800	C18—C19	1.400 (3)
C6—C24	1.508 (3)	C19—C20	1.382 (3)
C6—C7	1.575 (3)	C20—C21	1.381 (4)
C6—H6	0.9800	C20—H20	0.9300
C7—N1	1.461 (2)	C21—C22	1.362 (5)
C7—C8	1.507 (3)	C21—H21	0.9300
C7—H7	0.9800	C22—C23	1.387 (4)
C8—C9	1.390 (3)	C22—H22	0.9300
C8—C13	1.396 (3)	C23—H23	0.9300
C9—C10	1.382 (3)	C24—O1	1.198 (2)
C9—H9	0.9300	C24—O2	1.328 (3)
C10—C11	1.361 (4)	C25—C26	1.341 (6)
C10—H10	0.9300	C25—O2	1.463 (3)
C11—C12	1.371 (4)	C25—H25A	0.9700
C11—H11	0.9300	C25—H25B	0.9700
C12—C13	1.388 (3)	C26—H26A	0.9600
C12—H12	0.9300	C26—H26B	0.9600
C13—O4	1.380 (2)	C26—H26C	0.9600
			
N1—C1—C2	102.65 (19)	C15—C14—H14A	109.7
N1—C1—H1A	111.2	O4—C14—H14B	109.7
C2—C1—H1A	111.2	C15—C14—H14B	109.7
N1—C1—H1B	111.2	H14A—C14—H14B	108.2
C2—C1—H1B	111.2	C14—C15—C16	114.1 (2)
H1A—C1—H1B	109.2	C14—C15—H15A	108.7
C3—C2—C1	108.3 (2)	C16—C15—H15A	108.7
C3—C2—H2A	110.0	C14—C15—H15B	108.7
C1—C2—H2A	110.0	C16—C15—H15B	108.7
C3—C2—H2B	110.0	H15A—C15—H15B	107.6
C1—C2—H2B	110.0	C17—C16—C15	113.7 (2)
H2A—C2—H2B	108.4	C17—C16—H16A	108.8
C2—C3—C4	105.3 (2)	C15—C16—H16A	108.8
C2—C3—H3A	110.7	C17—C16—H16B	108.8
C4—C3—H3A	110.7	C15—C16—H16B	108.8
C2—C3—H3B	110.7	H16A—C16—H16B	107.7
C4—C3—H3B	110.7	O3—C17—C16	106.44 (18)
H3A—C3—H3B	108.8	O3—C17—H17A	110.4
N1—C4—C3	104.83 (18)	C16—C17—H17A	110.4
N1—C4—C5	106.03 (15)	O3—C17—H17B	110.4
C3—C4—C5	115.4 (2)	C16—C17—H17B	110.4
N1—C4—H4	110.1	H17A—C17—H17B	108.6
C3—C4—H4	110.1	O3—C18—C23	123.4 (2)
C5—C4—H4	110.1	O3—C18—C19	115.44 (18)
C19—C5—C6	114.87 (17)	C23—C18—C19	121.2 (3)
C19—C5—C4	114.30 (16)	C20—C19—C18	117.0 (2)
C6—C5—C4	105.27 (16)	C20—C19—C5	121.0 (2)
C19—C5—H5	107.3	C18—C19—C5	122.0 (2)
C6—C5—H5	107.3	C21—C20—C19	122.6 (3)
C4—C5—H5	107.3	C21—C20—H20	118.7
C24—C6—C5	112.41 (16)	C19—C20—H20	118.7
C24—C6—C7	116.88 (15)	C22—C21—C20	119.2 (3)
C5—C6—C7	102.34 (14)	C22—C21—H21	120.4
C24—C6—H6	108.3	C20—C21—H21	120.4
C5—C6—H6	108.3	C21—C22—C23	120.8 (3)
C7—C6—H6	108.3	C21—C22—H22	119.6
N1—C7—C8	117.63 (15)	C23—C22—H22	119.6
N1—C7—C6	106.88 (14)	C18—C23—C22	119.3 (3)
C8—C7—C6	118.99 (15)	C18—C23—H23	120.4
N1—C7—H7	103.7	C22—C23—H23	120.4
C8—C7—H7	103.7	O1—C24—O2	122.53 (19)
C6—C7—H7	103.7	O1—C24—C6	127.2 (2)
C9—C8—C13	117.10 (19)	O2—C24—C6	110.22 (17)
C9—C8—C7	124.01 (17)	C26—C25—O2	110.6 (3)
C13—C8—C7	118.87 (17)	C26—C25—H25A	109.5
C10—C9—C8	121.8 (2)	O2—C25—H25A	109.5
C10—C9—H9	119.1	C26—C25—H25B	109.5
C8—C9—H9	119.1	O2—C25—H25B	109.5
C11—C10—C9	119.8 (2)	H25A—C25—H25B	108.1
C11—C10—H10	120.1	C25—C26—H26A	109.5
C9—C10—H10	120.1	C25—C26—H26B	109.5
C10—C11—C12	120.5 (2)	H26A—C26—H26B	109.5
C10—C11—H11	119.8	C25—C26—H26C	109.5
C12—C11—H11	119.8	H26A—C26—H26C	109.5
C11—C12—C13	119.9 (2)	H26B—C26—H26C	109.5
C11—C12—H12	120.0	C7—N1—C1	119.67 (15)
C13—C12—H12	120.0	C7—N1—C4	103.34 (14)
O4—C13—C12	120.23 (19)	C1—N1—C4	106.37 (16)
O4—C13—C8	118.72 (18)	C24—O2—C25	116.4 (2)
C12—C13—C8	120.9 (2)	C18—O3—C17	120.56 (17)
O4—C14—C15	110.0 (2)	C13—O4—C14	118.34 (18)
O4—C14—H14A	109.7		
			
N1—C1—C2—C3	−22.2 (3)	O3—C18—C19—C5	−1.1 (3)
C1—C2—C3—C4	1.8 (4)	C23—C18—C19—C5	−179.8 (2)
C2—C3—C4—N1	19.5 (3)	C6—C5—C19—C20	−126.8 (2)
C2—C3—C4—C5	−96.7 (3)	C4—C5—C19—C20	111.3 (2)
N1—C4—C5—C19	150.07 (17)	C6—C5—C19—C18	54.1 (3)
C3—C4—C5—C19	−94.4 (2)	C4—C5—C19—C18	−67.7 (3)
N1—C4—C5—C6	23.1 (2)	C18—C19—C20—C21	−1.0 (4)
C3—C4—C5—C6	138.66 (19)	C5—C19—C20—C21	179.9 (2)
C19—C5—C6—C24	107.9 (2)	C19—C20—C21—C22	0.2 (4)
C4—C5—C6—C24	−125.50 (17)	C20—C21—C22—C23	0.4 (5)
C19—C5—C6—C7	−125.90 (18)	O3—C18—C23—C22	−179.1 (2)
C4—C5—C6—C7	0.73 (18)	C19—C18—C23—C22	−0.5 (4)
C24—C6—C7—N1	98.75 (19)	C21—C22—C23—C18	−0.3 (4)
C5—C6—C7—N1	−24.51 (18)	C5—C6—C24—O1	30.9 (3)
C24—C6—C7—C8	−37.5 (2)	C7—C6—C24—O1	−87.0 (3)
C5—C6—C7—C8	−160.75 (16)	C5—C6—C24—O2	−148.88 (17)
N1—C7—C8—C9	−74.5 (2)	C7—C6—C24—O2	93.2 (2)
C6—C7—C8—C9	57.2 (3)	C8—C7—N1—C1	58.7 (2)
N1—C7—C8—C13	107.4 (2)	C6—C7—N1—C1	−78.3 (2)
C6—C7—C8—C13	−120.91 (19)	C8—C7—N1—C4	176.60 (16)
C13—C8—C9—C10	2.4 (3)	C6—C7—N1—C4	39.67 (17)
C7—C8—C9—C10	−175.7 (2)	C2—C1—N1—C7	151.1 (2)
C8—C9—C10—C11	−0.3 (4)	C2—C1—N1—C4	34.8 (3)
C9—C10—C11—C12	−1.7 (4)	C3—C4—N1—C7	−161.31 (18)
C10—C11—C12—C13	1.5 (3)	C5—C4—N1—C7	−38.75 (18)
C11—C12—C13—O4	−175.03 (19)	C3—C4—N1—C1	−34.5 (2)
C11—C12—C13—C8	0.7 (3)	C5—C4—N1—C1	88.11 (18)
C9—C8—C13—O4	173.20 (17)	O1—C24—O2—C25	5.0 (3)
C7—C8—C13—O4	−8.6 (3)	C6—C24—O2—C25	−175.1 (2)
C9—C8—C13—C12	−2.6 (3)	C26—C25—O2—C24	160.8 (4)
C7—C8—C13—C12	175.65 (18)	C23—C18—O3—C17	−11.7 (3)
O4—C14—C15—C16	−67.6 (3)	C19—C18—O3—C17	169.63 (19)
C14—C15—C16—C17	160.5 (2)	C16—C17—O3—C18	−173.41 (19)
C15—C16—C17—O3	−68.4 (3)	C12—C13—O4—C14	−63.7 (3)
O3—C18—C19—C20	179.87 (19)	C8—C13—O4—C14	120.5 (2)
C23—C18—C19—C20	1.1 (3)	C15—C14—O4—C13	−100.1 (3)

### Hydrogen-bond geometry (Å, º)

Cg3 and Cg4 are the centroids of the C8–C13 and C18–C23 rings, respectively.

**Table:**

*D*—H···*A*	*D*—H	H···*A*	*D*···*A*	*D*—H···*A*
C11—H11···O4^i^	0.93	2.60	3.419 (3)	148
C17—H17*B*···*Cg*4^ii^	0.97	2.97	3.817 (3)	146
C22—H22···*Cg*3^iii^	0.93	2.94	3.770 (3)	150

Symmetry codes: (i) −*x*+1, *y*−1/2, −*z*+1/2; (ii) −*x*+1, −*y*+1, −*z*; (iii) *x*, −*y*+1/2, *z*−1/2.
